# Advances in Pancreatic Ductal Adenocarcinoma Treatment

**DOI:** 10.3390/cancers13215510

**Published:** 2021-11-03

**Authors:** Eric M. Anderson, Shant Thomassian, Jun Gong, Andrew Hendifar, Arsen Osipov

**Affiliations:** 1 Department of Radiation Oncology, Memorial Sloan Kettering Cancer Center, New York, NY 10065, USA; anderse@mskcc.org; 2 Department of Medicine, Samuel Oschin Comprehensive Cancer Institute, Cedars Sinai Medical Center, Los Angeles, CA 90048, USA; sthomassian15@email.mmc.edu (S.T.); jun.gong@cshs.org (J.G.); andrew.hendifar@cshs.org (A.H.)

**Keywords:** pancreatic ductal adenocarcinoma, tumor microenvironment, immunotherapy, immune checkpoint, CTLA-4, PD-1, PDL-1, vaccine, targeted therapy, CXCR4, PARPi, CAF

## Abstract

**Simple Summary:**

Pancreatic cancer remains one of the most challenging malignancies to treat with standard approaches. Emerging treatment approaches incorporating innovative surgical techniques and novel systemic therapies may help to improve outcomes for pancreas cancer patients. Although immunotherapy has proven to be less effective for the treatment of pancreas cancer relative to more immunogenic tumor types, multiple immune system stimulating agents are under active investigation for pancreatic ductal adenocarcinoma, either alone or in combination with other therapeutic agents. The tumor microenvironment of pancreatic cancer is also an attractive therapeutic target given that it is believed to be highly immunosuppressive and encapsulated by a dense stroma. This review article aims to summarize pre-clinical and clinical studies, including ongoing clinical trials, which attempt to incorporate novel treatment approaches for pancreas cancer. Emerging treatment approaches may help to significantly improve outcomes for this tough to treat disease.

**Abstract:**

Pancreatic Ductal Adenocarcinoma (PDAC) is one of the deadliest malignancies among all cancers. Despite curative intent, surgery and the use of standard cytotoxic chemotherapy and radiation therapy, PDAC remains treatment-resistant. In recent years, more contemporary treatment modalities such as immunotherapy via checkpoint inhibition have shown some promise in many other malignancies, yet PDAC still eludes an effective curative treatment. In investigating these phenomena, research has suggested that the significant desmoplastic and adaptive tumor microenvironment (TME) of PDAC promote the proliferation of immunosuppressive cells and act as major obstacles to treatment efficacy. In this review, we explore challenges associated with the treatment of PDAC, including its unique immunosuppressive TME. This review examines the role of surgery in PDAC, recent advances in surgical approaches and surgical optimization. We further focus on advances in immunotherapeutic approaches, including checkpoint inhibition, CD40 agonists, and discuss promising immune-based future strategies, such as therapeutic neoantigen cancer vaccines as means of overcoming the resistance mechanisms which underly the dense stroma and immune milieu of PDAC. We also explore unique signaling, TME and stromal targeting via novel small molecule inhibitors, which target KRAS, FAK, CCR2/CCR5, CXCR4, PARP and cancer-associated fibroblasts. This review also explores the most promising strategy for advancement in treatment of pancreatic cancer by reviewing contemporary combinatorial approaches in efforts to overcome the treatment refractory nature of PDAC.

## 1. Introduction

Pancreatic ductal adenocarcinoma, PDAC, which accounts for greater than 90% of pancreatic tumors, remains a dreadful disease characterized by poor prognosis. Pancreas cancer accounts for 3% of all cancers in the Unites States (US) and 7% of all cancer deaths. Accordingly, the American Cancer Society estimates approximately 60,430 Americans will be diagnosed with pancreatic cancer and about 48,220 will die from pancreatic cancer in 2021 [1,2]. Even more concerning is that it is estimated by the year 2030, pancreatic cancer will become the second leading cause of cancer-related death in the United States. Current standard of care treatments including surgery, chemotherapy and radiation therapy remain incompletely effective treatment strategies as PDAC has proven to be highly resistant to these modalities Additionally, whereas surgical resection has the potential of offering a curative treatment in early stages, nearly 80% of those who undergo surgery have disease recurrence, and PDAC typically presents in late stages as symptoms are non-specific. In recent years, advances in the field of immunotherapy and targeted therapies have led to a shift in treatment paradigms for cancer care. These newfound insights into the intricate interactions between the immune system and cancer cells has opened new avenues of treatment, such as checkpoint blockade via targeting of CTLA-4 and PD-1/PDL1, with the goal of recruiting and promoting immune cells to eliminate tumor cells. However, the efficacy of these novel treatments have been limited to certain tumor types that are considered highly ‘immunogenic’ including melanoma and lung cancer, while PDAC has been generally resistant to immunotherapy [3,4,5,6,7]. Although tumor mutational burden has been associated with response to immune checkpoint inhibitors in multiple cancer types, PDAC less commonly demonstrates high mutation burden [8]. Additionally, the tumor cells of PDAC have also been found to be surrounded by a desmoplastic stroma with adaptive responses shaping the tumor microenvironment (TME) to enhance tumor survival, proliferation, and metastasis. Additionally, communication between cells, such as carcinoma associated fibroblasts or immunosuppressive myeloid cells or regulatory T cells of the TME possess the capacity to effectively silence immune response, contributing further to treatment resistance. Research into these cell types in the TME of PDAC have shed light on the failures of current treatment approaches in PDAC. Appropriate exploitation of these pathways or other well-known pancreas cancer potential molecular targets like KRAS, via a combinatorial, the TME can be leveraged to convert a tumor from ‘non-immunogenic’ to one that may be effectively targeted by immune cells, thereby improving treatments and outcomes for patients with PDAC. Additionally, even more traditional approaches in pancreatic cancer such as surgery, have continued to develop new advances to improve outcomes and clinical responses. In this review, we will explore advances in the treatment of pancreatic cancer, from surgical approaches to TME targeting, immunomodulation, immunotherapy, molecular targeting, and novel combinatorial strategies (Table 1).

## 2. Surgical Approaches

Although significant advances have been made in exploring and developing a range of novel systemic therapies for the treatment of pancreas cancer, surgical resection remains an essential component of curative intent treatment. Accordingly, innovation in surgical approaches may help to improve long-term outcomes for pancreas cancer patients. Modern advances in technical approaches to multiple aspects of pancreatic cancer surgery have been pursued in recent years. For patients with borderline resectable and locally advanced pancreas cancer, surgical resection, particularly an R0 resection, and optimal surgical approach may have the best chance of improving patient outcomes. In this patient population, techniques that optimize surgical resection and vessel reconstruction may lead to the most significant improvements in outcomes [9]. Multiple studies have demonstrated clear survival benefit associated with margin negative resection [10,11].

Innovations in surgical techniques have allowed for more aggressive resection combined with reconstruction of adjacent blood vessels. For example, the Appleby procedure is a relatively well-established surgical technique that is particularly helpful for patients with locally advanced disease. This procedure involves en bloc resection of the celiac trunk and common hepatic artery along with distal pancreatosplenectomy. Data suggest that the Appleby procedure is associated with a lower risk of hemorrhage [12]. Further studies are needed to assess for potential improvement in clinical outcomes associated with use of the Appleby procedure.

Another emerging area of surgical innovation in pancreas cancer treatment is the concept of the mesopancreas. A well-established body of literature has established the benefit of total mesorectal excision for the treatment of rectal cancer. This success has led surgeons to consider a similar anatomic construct for the treatment of pancreas cancer. The mesopancreas has been defined as a firm and well-vascularized structure extending from the posterior surface of the pancreatic head to behind the mesenteric vessels. Given that the mesopancreas has been found to be a common site of positive resection margins for patients with pancreas cancer involving the pancreatic head, total mesopancreas resection has been considered as a potential approach to improving clinical outcomes. A recent single institutional analysis demonstrated that total mesopancreas resection was associated with a significantly higher rate of margin negative resection and number of lymph nodes resected, as well as a lower recurrence rate and longer disease free survival [13]. A more recent larger single institutional study demonstrated a nearly 50% margin negative resection rate associated with total mesopancreas resection with a nearly 80% rate of mesopancreas fat infiltration by tumor [14]. In multivariable analysis in this study, only margin negative resection was an independent prognostic factor, and local recurrence was only identified in approximately 10% of patients with margin negative resection. This approach has been further refined to incorporate a so-called “no-touch” technique. The no-touch isolation technique has been pursued for other disease sites, including colon and eye cancer surgery, given concern that physical manipulation of the involved tumor may result in tumor cell shedding into adjacent tissues and blood vessels. Accordingly, a no-touch technique was developed for total mesopancreas excision with pancreaticoduodenectomy in an effort to reduce the risk of pancreas cancer cell shedding into the portal vein, retroperitoneum, peritoneal cavity, and other adjacent anatomic structures. A retrospective study evaluating clinical outcomes using this no-touch technique demonstrated an approximate 70% rate of margin negative resection with associated rates of 5-year overall and recurrence-free survival of 42% and 32%, respectively, among patients with margin negative resection [15].

Irreversible electroporation (IRE) is a technique that relies on high voltage microsecond electrical pulses to induce cell membrane porosity leading to cell death. This technique has been applied intraoperatively in patients with locally advanced pancreas cancer. Compared with thermal ablation techniques, including radiofrequency ablation, microwave ablation, and cryoablation, IRE has the benefits of more well-defined areas of tumor damage with relative sparing of adjacent connective tissues and minimal impact of blood flow on its effects. Clinical studies have demonstrated that use of IRE results in significant improvements in both local and distant progression-free survival, as well as overall survival [16].

Increasing emphasis has also been placed on clinical selection and optimizing medical management for patients being considered for surgical resection for pancreas cancer. Although advances in surgical techniques have resulted in improved post-operative mortality for patients undergoing pancreaticoduodenectomy, associated morbidity remains high. Sarcopenia is an emerging predictor of post-operative morbidity after pancreas cancer resection. A recent retrospective analysis demonstrated that sarcopenia is associated with both advanced age and preoperative albumin levels and was a significant predictor of post-operative complications [17]. Furthermore, body mass index (BMI) has been evaluated as a predictor of outcomes after resection for pancreas cancer. A large retrospective analysis demonstrated that lower BMI and serum albumin levels were associated with longer post-operative hospital stay and worse survival [18].

Despite advances in surgical techniques, treatment approaches, and patient optimization, disease recurrence and treatment resistance mediated by the TME and stroma of pancreas cancer remain both a serious challenge and simultaneously an avenue for therapeutic targeting as well as novel treatment advances.

## 3. The TME of Pancreatic Caner

**Figure 1:**
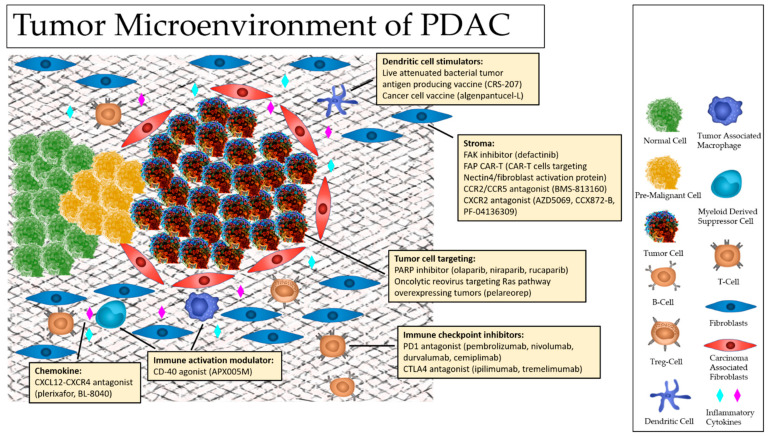
Pancreatic Ductal Adenocarcinoma (PDAC) Tumor Microenvironment.

Normal cells live in harmony with their surrounding environment, communicating with stromal cells in ways that support survival and healthy growth even in the face of pathogens. In comparison, cancer cells have the capacity to interact with immune cells and stromal cells enhance their survival and growth. By manipulating cellular activity and selectively altering cell populations, cancer cells provide themselves opportunities to thrive and rapidly proliferate. Typically, this is accomplished by tipping the balance between pro-tumor and anti-tumor forces within the TME. This adaptive characteristic noted in PDAC plays a significant role in tumor development, serves as a primary factory in immune escape, and has been an obstacle in treatment strategies [19]. In order to understand what alterations have been made and their implications on tumorigenesis and survival in PDAC, we must first consider the key players involved. The TME of pancreatic cancers encompasses normal cells, tumor cells, pre-malignant cells, diverse lines of stromal cells, immune cells, the extracellular matrix (ECM), cytokines, and a vast array of communication pathways between them [18]. Among the cells of the TME, B-cells, T-cells, dendritic cells (DCs), tumor-associated macrophages (TAMs), carcinoma-associated fibroblasts (CAFs), and myeloid-derived suppressor cells (MDSCs) have been noted to play more active roles in carcinogenesis and treatment resistance (Figure 1). Additionally, dependent on the inflammatory milieu at the time, the complex interplay between these cells and intercellular pathways of the TME have been found to result in diverse effects ranging from restraining cancer proliferation, promoting tumoricidal activity, or facilitating tumor development and progression.

Various studies in PDAC have shown an upregulation of regulatory T-cells (Tregs) and immature DC’s infiltrating resected tissue regardless of stage [19,20,21,22]. These cells can cause suppression of T-cell activation and anti-tumor immune response in PDAC [23]. In an immunologically unfavorable state, the TME is inundated with immunosuppressive cells paired with a lack of high-quality T-effector cells. Additionally, PDAC is characterized by alterations and increase in the diversity of myeloid phenotypes, which can be immunosuppressive, including myeloid-derived cells, predominantly MDSCs, TAMs and neutrophils in varying stages of differentiation [24]. The presence of MDSCs, TAMs and other subsets of myeloid cells have been implicated in the promotion of carcinogenesis, metastasis, and poor prognosis [25,26,27]. Recent studies have shown the tumor promoting nature of MDSCs, both polymorphonuclear and monocytic, whereas TAMs have shown to poses both pro-tumor and anti-tumor properties [28,29]. Dependent on phenotype expression, macrophages may differentiate into pro-inflammatory “M1” or anti-inflammatory “M2” versions [30]. In healthy tissue, the M1 and M2 phenotypes exist in equilibrium. However, in the setting of PDAC and other progressive cancers, the ratio becomes biased toward the M2 phenotype [31,32,33]. In contrast, the M1 phenotype appears to be the dominant population noted in regressing tumors. This variance in phenotypic expression is attributed to signals within the TME from various cytokines. The upregulation of cytokine expression can have serious consequences within the TME. Expression of certain ‘pro-tumor’ cytokines such as tumor necrosis factor-α (TNF-α), interlukin-6 (IL-6), and IL-8 have been found to promote tumor growth and metastasis. While other ‘anti-tumor’ cytokines, like IL-4, IL-13 and IL-10 have been documented to support an anti-inflammatory environment and suppress adaptive immune responses [33,34,35].

The presence of tumor promoting cells and cytokine-related inflammatory changes are thought to significantly contribute to the limited success of modern treatment strategies [36,37,38,39]. Intracellular pathways influenced by tumor cells function as the activators and promoters of the TME’s capacity for tumor escape mechanisms and immune tolerance. Key players of note include signaling via Kirsten rat sarcoma viral oncogene (KRAS). Mutation in KRAS, found to be present in over 90% of PDAC, is known to promote tumor carcinogenesis [40]. Functioning as GTPases, KRAS oncogenes trigger wide-reaching downstream effects, including the activation of various TME cells, influencing the expression of immune mediators [41]. These pathways include but are not limited to the downregulation of major histocompatibility complex (MHC class 1), thereby reducing the ability of T-cells to recognize antigen, upregulation of MDSCs, inducing recruitment of Tregs, and promoting stromal remodeling by CAFs and the ECM [42,43]. In cancer, the ECM serves roles much greater than a simple scaffold for cells, associated lymphatic tracts, and vascular systems. While immune cells and tumor cells are the sources of cytokines, growth factors, and various relevant pro-tumor/anti-tumor molecules, their effects are potentiated via interactions with the ECM. Various relationships between the ECM and TME have been found to facilitate cancer survival and progression. The ECM of PDAC generates a thick stromal compartment with reduced vascularization, which poses challenges in drug penetration. PDAC characteristically produces an intense desmoplastic reaction including a well-developed stromal component comprising as much as 60–90% of the tumor mass and significant hyaluronic acid (HA) [44,45]. This impenetrable physical barrier and a source of reactive oxygen species (ROS), forms complex signaling axes between neoplastic, immune and stromal cells that regulate cancer growth, manipulate immune surveillance, and limit the efficacy of immunotherapy [46,47,48,49]. In addition to these defensive features, the ECM has the capacity to control the TME via crosstalk between the ECM and intracellular pathways, such as those involving focal adhesion kinase (FAK), which upregulated in multiple malignancies and is known to alter intracellular and intercellular processes involving cellular adhesion, apoptosis and survival [50]. In the case of PDAC, tumor cells may manipulate the ECM to FAK signaling, regulating differentiation and motility to promote a pro-tumor, anti-inflammatory environment [51]. Activation of FAK via ECM-derived signals has been shown to spawn the migration of pro-tumor chemokines, Tregs, and TAMs, leading to an increased immunosuppressive TME, evasion of anti-tumor immunity, and promotion of metastasis [52,53].

Hypoxia is also believed to play a significant role in the TME of pancreas cancer. Hypoxia occurs in this environment as a result of increased oxygen consumption in the setting of rapid tumor cell proliferation and poor vascularization in a dense desmoplastic stromal environment. The cellular physiologic response to hypoxia is driven primarily by hypoxia-inducible factors (HIFs), which serve as active transcription factors under hypoxic conditions. Corresponding gene transcription has downstream effects on multiple pathways involved in tumorigenesis and cancer progression, including metabolic reprogramming, autophagy, maintenance of stemness, epithelial to mesenchymal transition (EMT), angiogenesis, tumor invasion, migration, and metastasis [54].

All of the elements of the TME, from immunosuppressive myeloid cells to stromal fibroblasts, as well as a lack of high quality effector T cell, pose a significant obstacle to traditional cytotoxic therapy efficacy. However, they are also rational targets for advances in treatment. Immunomodulation, in the TME and beyond, may also prove to be an effective therapeutic strategy in PDAC.

## 4. Immunotherapy

Immunotherapy can help to alleviate immunosuppressive changes observed in the TME. By reprogramming the cells of the TME, immunotherapy has the potential to trigger anti-tumor immune response. There have been a variety of immune-modulating therapies incorporated into cancer treatment in recent years, including the use of monoclonal antibody checkpoint inhibitors and cancer vaccines.

Since FDA approval of the first immune checkpoint inhibitor, ipilimumab, the use of checkpoint inhibitors has proved to be among the most promising breakthroughs in modern cancer treatment. Ipilimumab, initially approved for treatment of stage III/IV melanoma, achieves therapeutic action by blocking the cytotoxic T lymphocyte antigen 4 (CTLA-4) from binding to its ligands B7.1 and B7.2, effectively functioning as an inhibitor of IL-2 production and T-cell differentiation [55]. CTLA-4, when active, signals to cellular pathways leading to profound changes in immunosuppressive function of T-cells, as well as the production of regulatory cytokines and inhibitory proteins [56,57,58]. Although CTLA-4 blockade was successful in melanoma, when used as single agents in stage III/IV PDAC, they did not show efficacy [7].

As research in immunotherapy advanced, various other immune checkpoints were found to be of significant clinical importance, including most notably the programmed death receptor-1 (PD-1) and its ligand PD-L1. In 2014, the PD-1/PD-L1 blocking antibodies pembrolizumab and nivolumab were approved for the treatment of stage III/IV melanoma after showing efficacy [59,60]. Since then, PD-1/PD-L1 blocking agents have shown significant anti-tumor activity in multiple tumor types, including non-small cell lung cancer and gastrointestinal cancers with mismatch repair deficiencies [61,62]. However, PDAC has shown resistance to therapeutic approaches inhibiting PD-1/PD-L1 pathways. When used as a single agent in locally advanced or metastatic pancreatic cancer, PD-1 checkpoint inhibition proved ineffective with no improvement in survival [7,63]. This lack of clinical response may be due to the makeup of cells within the TME. Cancers with strong response to checkpoint inhibition tend to have a TME with numerous high-quality T-cells and effector lymphocytes [64] while the TME of PDAC is predominantly occupied by immunosuppressive cells and is nearly devoid of pro-inflammatory anti-tumor cells [65,66].

Resistance to single checkpoint inhibition has been well documented in PDAC. This phenomenon tends to occur due to upregulation of secondary compensatory pathways within the TME. In efforts to overcome this burden, studies investigating the use of dual checkpoint inhibitors have been conducted with the hope of identifying synergistic effects between the anti-PD-L1 antibody durvalumab used in combination with the anti-CTLA-4 antibody tremelimumab. Unfortunately, as demonstrated in a phase II trial, dual checkpoint inhibition failed to show therapeutic response in PDAC, and subsequent trials has continued to explore this approach [67].

In contrast to the blockade of pathways, an alternative approach to reprograming the TME is via the use of monoclonal antibodies as agonists. Among the targets of agonist therapy is CD-40, which is a costimulatory molecule present on antigen-presenting cells such as macrophages. In PDAC mouse models, CD-40 agonist enhanced the TME by promoting inflammatory action [68]. When activated, CD-40 can facilitate a more favorable TME by causing an upregulation of TAMs expressing MHC class II (MHC-II), recruitment of T-cells, and degradation of stromal barriers [68]. The success of CD-40 agonist treatment in mouse models has established this approach as a potential therapeutic target. Currently, CD-40 agonist CP-870,893 is being studied in early phase trials in combination with gemcitabine for patients with PDAC. Additionally, CD-40 agonist monoclonal antibody APX005M is being investigated in phase 1 trials for pancreatic cancer. Data from a randomized study of standard of care gemcitabine and nab-paclitaxel with or without nivolumab reported at the 2021 ASCO Annual Meeting demonstrated a significant improvement in one year overall survival (48% vs. 35%, *p* = 0.06). This study also showed a borderline statistically significant improvement in 1 year overall survival (48% vs. 35%, *p* = 0.06) along with, and increase of activated myeloid dendritic cells and tumoral M1 macrophages associated with the use of standard of care chemotherapy with or without sotigalimab [69].

Another method of augmenting cells of the TME of PDAC is to enhance anti-tumor immune activity is using therapeutic vaccines. Like viral vaccinations, these vaccines can function by administering pancreatic tumor antigens to stimulate patient anti-tumor immune response by providing an opportunity for the immune system to recognizing variations between tumor cells and normal pancreatic cells. Current research in the field of therapeutic vaccines is studying the utility of numerous types of vaccines, most notably whole-cell vaccines and antigen-specific vaccines. Among the whole-cell therapeutic vaccines, GVAX was developed for the treatment of PDAC and consists of two allogenic cell lines that stimulate therapeutic action by inciting immune response against a wide range of PDAC antigens, ultimately leading to secretion of granulocyte-macrophage colony stimulating factor [70,71,72]. Given two weeks prior to surgical resection, 84.6% of GVAX patient samples express increased tertiary lymphoid aggregates when compared to GVAX-naive patients. Studies evaluating the efficacy of GVAX in PDAC have shown enhanced T-cell proliferation and differentiation, which was associated with longer progression-free survival in a subset of patients [73,74]. Additional studies combining GVAX with low dose cyclophosphamide (Cy) show a more durable response when compared to GVAX alone [73]. Although lymphoid aggregates are present when GVAX is administered with or without Cy, the combination with Cy allowed for enhanced effect of cancer vaccines by providing a more favorable TME nearly devoid of Tregs [70]. These results indicate that GVAX successfully induced adaptive immune response in patients with PDAC. Furthermore, GVAX was found to induce an upregulation of PD-1/PD-L1 [70]. In contrast to tumors which have responded well to PD-1/PD-L1 checkpoint blockade, PDAC is known to typically express low levels of PD-L1. However, tumor cells of patients who received GVAX demonstrated moderate levels of PD-L1 [70]. These results taken together suggest that GVAX may convert the “non-immunogenic” PDAC TME into one that is “immunogenic”, creating an optimal environment for checkpoint inhibitor therapy. In a preclinical study, the combination of GVAX and PD-1 inhibitors significantly improved survival in tumor-bearing mice when compared to GVAX or anti PD-1 therapy used as single agents [75]. Moreover, a pilot study evaluating the efficacy of GVAX in combination with ipilimumab in patients with previously treated PDAC found a 20% objective response [76]. Data collected from these studies support the utility of combination GVAX and checkpoint inhibition in the treatment of PDAC.

Algenpantucel-L is another whole cell vaccine under investigation. This vaccine is composed of irradiated allogenic cancer lines that have been modified to express murine α-1,3-galactosyltransferase, leading to accumulation of alpha-galactosyl residues on the cell surface [77]. In theory, immunization with algenpantucel-L would yield a production of antibodies targeting alpha-galactosyl, leading to enhanced anti-tumor response. As expected, studies have shown a robust response to immunization with this drug leading to hyperacute rejection and lysis of alpha-galactosyl epitopes. Although this therapeutic vaccine was shown to be well-tolerated in phase II trials, when studied in the phase III IMPRESS trial combining algenpantucel-L with current standard of care treatment, there was no observed improvement of overall survival with the use of this agent [78].

More recently, researchers have been able to further identify pancreatic tumor antigens, and peptide vaccines specifically targeting these antigens are being developed. Among these are the KRAS peptide vaccines for the treatment of pancreatic cancer. An estimated 90% of patients with pancreatic cancer have mutant KRAS specific to tumor cells [79,80,81,82]. However, treatment with peptide vaccines faces serious challenges as peptide vaccines must be matched to patient HLA type, immune evasion occurs frequently, and treatment must be administered with immune adjuvants or be carried by vectors to elicit response [83]. Another target identified for antigen-specific vaccines is mesothelin. After studies with GVAX found mesothelin-specific immune response in patients correlated with increased survival, mesothelin was investigated as a potential candidate for protein-specific vaccines. CRS-207 is a recombinant live-attenuated vaccine developed to enhance expression of mesothelin. Using Listeria monocytogenes as a vector to secrete tumor antigen into the cytosol of infected APCs, CRS-207 was shown to illicit both innate and adaptive immunity to antigens [84,85]. In a phase 2 trial comparing GVAX + Cy to combination GVAX/Cy + CRS-207 in patients with metastatic PDAC, overall survival was improved from 3.9 months in the GVAX + Cy arm to 6.1 months with GVAX/Cy + CRS-207. When doses were increased from 1 to at least 3, overall survival improved from 4.6 months to 9.7 months with GVAX/Cy + CRS207 [86]. This dose-dependent improvement in survival further supports the efficacy of therapeutic vaccines in the treatment of pancreatic cancer. An emerging area of cancer vaccine research is the use of neoantigen vaccines. A small clinical study recently found that the use of neoantigen vaccines for solid malignancies was safe and induced an immune response [87]. Recently, an early phase study conducted in China evaluating personalized peptide neoantigen vaccine against pancreatic cancer had shown promise, where patient was found to have 21 month OS and increase in T cell clonality, attributed the clinical success to T cell response [88]. Overall, further studies are needed to assess the clinical efficacy of this approach in greater detail, but early findings appear promising. Beyond immunotherapeutic approaches designed to target gene-specific alterations, such as KRAS, in PDAC cells, a wide range of molecularly targeted therapies have been developed for the treatment of pancreatic cancer.

## 5. Targeted Therapies

In addition to the paradigm shifting success of immunotherapy, over the last decade, molecular targeted therapies have also emerged as promising treatments for a range of cancer types. By directly targeting molecular pathways including those involved in tumor growth, carcinogenesis, and drug resistance, targeted therapies have offered a treatment modality that may serve as an adjuvant to chemotherapy, radiation therapy, and immunotherapy. This type of molecular targeting has been approached with the use of multiple types of agents including small molecule inhibitors (SMIs), which have a significantly smaller molecular size and weight than antibodies. SMIs have advantages in oral bioavailability, high tumor penetration, and relatively favorable toxicity profiles secondary to shorter half-life [89]. While monoclonal antibodies as single agents or in combination have shown to be effective for some tumor types, they have not demonstrated efficacy in PDAC and are not without risk of serious toxicities [90]. The lack of efficacy of antibody-based immunotherapies in PDAC may be related to the associated dense stromal compartment of the TME. Most antibodies have difficulty traversing the ECM and are limited to cell surface-level interactions. In contrast, SMIs can readily bypass ECM barriers and be made to target intracellular pathways [91]. Targeting cellular pathways can have significant downstream effects. By selectively targeting intercellular pathways, SMIs can manipulate nuclear, intracellular, and intercellular activity to potentially overcome the immunosuppressive TME.

FAK is among the intracellular pathways that have shown promise as a target in cancer treatment, in addition to other well-known signaling molecules including RAS-RAF-MEK1/2-ERK1/2, CCR2/CCR5, and CXCR4. Often upregulated in malignancies, FAK elicits effects including but not limited to, altering intracellular and intercellular processes involving cellular adhesion, apoptosis and survival [50]. FAK plays a role in tumor cell signaling as well as in stromal cells within the TME, including CAFs. The high activity of CAFs within the TME has been implicated in tumor growth, angiogenesis, metastasis and drug resistance [92]. Presence of CAFs has been associated with suppression in the cytotoxic capacity of CD 8 + T-cells, and T-cell dysfunction in addition to influencing the activity of MDSCs, TAMs, and Tregs [92,93,94]. However, studies with SMIs targeting FAK have shown that FAK inhibition may reduce the density of CAFs within the TME, decrease immunosuppressive MDSCs, TAMs, and Tregs, and in turn increase the presence and activity of CD 8 + cytotoxic T-cells [53,95]. An additional benefit of FAK inhibition is its ability to downregulate cancer stem cells (CSCs). CSCs confer tumors the potential to generate more cells with heterogeneous differentiation, leading to resistance to therapy, recurrence, and metastasis [96,97]. Because of its role as a potential TME master regulator, FAK is being investigated in multiple clinical trials.

Recently, a Phase I/II study of the combination of gemcitabine, pembrolizumab and defactinib in metastatic PDAC showed the combination of anti-PD1 pembrolizumab and FAKi defactinib with chemotherapy to be safe, without significant increase in toxicities compared to what we know with chemotherapy [98]. Nevertheless, due to the limited tissue size from biopsy and the single arm design, the correlative studies in this clinical trial are limited besides showing an increased CD8^+^ and CD4^+^ T cell infiltration and a decreased TAM and Treg infiltration following the combination therapy. Currently, the sequential combination of chemotherapy, followed by anti-PD-1 and FAKi is being investigated in the neoadjuvant setting allowing for comprehensive analysis of the TME and the implications of FAKi on it (NCT03727880).

Selectively altering chemokine signaling pathways is another approach to targeted therapy made possible by using SMIs. Among these is the targeting of CCR2 and CCR5, which are expressed on MDCS and are known to function as a monocyte recruiting signals to the TME when bound to their ligands, CCL2/CCL5 [99]. Mouse models have demonstrated that CCR2/CCR5 inhibition can effectively restrict TAM infiltration, providing a more favorable anti-tumor balance within the TME. This effect has been thought to synergize with PD-1 inhibition, thereby further potentiating immunotherapeutic response [100]. Given these favorable early stage results, investigation of CCR2/CCR5 inhibition in PDAC using SMIs CCX872 and PF-04136309 has been underway. However, early phase trials using CXC872 as monotherapy or PF-04136309 with chemotherapy have yielded inconsistent results [101,102,103]. PF-04136309 used in combination with gemcitabine and paclitaxel was associated with pulmonary toxicities and was not found to be more efficacious than combination chemotherapy with gemcitabine and paclitaxel alone. Yet, when combined with FOLFIRINOX, CXC872 and PF-04136309 did show promising results. Further study of CCR2/CCR5 inhibition is ongoing using small molecule BMS-813160 in a phase II trial studying its utility when combined with chemotherapy or immunotherapy with nivolumab (NCT03184870).

While CCR2/CCR5 can regulate the density of TAMs in the TME, another signaling protein present on MDSCs, CXCR2, is known to control chemotaxis of tumor-associated neutrophils [104,105]. Though the role of tumor-associated neutrophils in carcinogenesis is not yet completely understood, their presence has been associated with poor prognosis in most cancers [106]. When studied in mouse models, CXCR2 knock out led to a reduction of neutrophils and a proportional suppression of tumor growth [107]. In further investigation, it was found that inhibition of CXCR2 can have other significant effects in PDAC, such as limiting tumor metastasis, creating a favorable T-cell balance within the TME, enhance response to chemotherapy, and to anti PD-1 therapy, and extending overall survival [108,109]. An ongoing phase I/II study of the CXCR2 antagonist CCX872-B along with gemcitabine/nab-paclitaxel is assessing the safety and efficacy of this combination (NCT02732938). A similar study is evaluating CCX872-B, which is a CXCR2 antagonist, in combination with FOLFIRINOX in a phase I study of patients with PDAC (NCT02345408).

Certain solid tumors have shown to be deficient in various DNA repair mechanisms, such as BRCA mutations in breast cancer. These deficiencies have been exploited with platinum-based agents with some success. However, tumor cells may mitigate effects of these treatments by utilizing poly-adenosine-diphosphate-ribose polymerase (PARP) an enzyme which possesses the molecular function of detecting damage in DNA and promoting repair [110]. The use of PARP inhibitors (PARPi) is relatively new. However, after showing efficacy in trials olaparib, niraparib, and rucaparib have been approved for the treatment of ovarian cancer in the presence of BRCA mutation. Given we find mutated BRCA1/BRCA2 genes in a significant proportion of patients with familial pancreatic cancer, the utility of these PARPi have been explored in PDAC trials [111,112,113]. In a phase II trials of olaparib and rucaparib, pancreatic cancer patients with BRCA1/BRCA2 mutations showed response rates of 22% and 16%, respectively [114,115]. The successes of phase II studies led to the phase III POLO trial. In this landmark study, patients with metastatic pancreatic cancer with confirmed BRCA1/BRCA2 were pre-treated with platinum-based chemotherapy followed by either maintenance olaparib or placebo. Maintenance therapy was associated with significantly improved progression-free (hazard ratio [HR], 0.53; 95% confidence interval [CI], 0.35–0.82; *p* = 0.004) but not overall (HR 0.83; 95% CI 0.56–1.22; *p* = 0.35) survival [116].

KRAS is another significant therapeutic target in PDAC given the very high associated rate of mutation [117]. Historically, KRAS-targeted therapies have focused on indirect approaches at impacting the pathway, including proteins that facilitate KRAS association with the cell membrane, KRAS-dependent metabolic pathways, KRAS effector protein signaling, and synthetic lethal protein interactions. The development of RAS-binding small molecule inhibitors has also been accelerated in recent years, and a recent publication described clinical efficacy of a KRAS inhibitor for the treatment of non-small-cell lung cancer, leading to FDA approval [118]. Furthermore, multiple studies have demonstrated that the extracellular regulated kinase (ERK) mitogen-activated protein kinase (MAPK) pathway plays a key role in KRAS-dependent tumor initiation, progression, and maintenance. Studies to date have generally demonstrated limited efficacy of MEK1/2 selective inhibitors in RAS mutant cancers, and trametinib specifically did not demonstrate clinical efficacy in KRAS mutant PDAC [119]. Ongoing early stage clinical trials are also evaluating ERK inhibitors in combination with chemotherapy for PDAC. The clinical efficacy of second-generation RAF inhibitors is also being explored in KRAS mutant PDAC.

Multiple hypoxia-based therapeutic strategies have also been evaluated for PDAC. For example, metronomic gemcitabine has been shown to improve perfusion and reduce hypoxia in PDAC, in part due to associated functional blood vessel density. Additional vascular normalizing agents, including cediranib and nelfinavir, are also being evaluated in ongoing phase I/II clinical trials [56]. Furthermore, cyclooxygenase-2 (COX-2) inhibition can improve the efficacy of anti-VEGF treatment by reversing hypoxia-induced EMT [120]. Novel therapeutic approaches are also being developed to target HIF signaling pathways, including inhibitory siRNA, a CRISPR/Cas9 model that downregulates HIF-1α, and the selective HIF-1α inhibitor PX-478. Lastly, ongoing studies are also evaluating the potential therapeutic benefit of photodynamic therapy, which relies on light or sound waves, nanoparticles, and oxygen to generate oxygen free radicals, which damage PDAC cells and have a cytotoxic effect [121].

The peritumoral stroma is a specific component of the PDAC TME for which targeted therapeutics may help to significantly improve patient outcomes.

## 6. Stromal Targeting

The cancer stromal environment plays a key role in cancer cell survival and tumor growth in patients with PDAC. A number of molecular pathways that play a role in the cancer stromal environment have been identified as potential therapeutic targets for PDAC. One example is the CXCL12-CXCR4 chemokine signaling pathway. CXCR4, when bound to its ligand CXCL12, can manipulate the TME via crosstalk between stromal cells and immune cells [122]. Inhibition of this pathway has shown promising results in pre-clinical studies of PDAC. In mouse models, CXCR4 inhibitors BL-8040 and AMD3100 have promoted T cell access into the TME along with increasing sensitivity to PD-1/PD-L1 blocking therapy [123,124]. The efficacy of BL-8040 has been documented in the setting of melanoma, neuroblastoma, breast, and lung cancer. Through these studies, it was discovered that BL-8040 can promote mobilization of high-quality T-cells, B cells, natural killer cells. A phase II study in PDAC showed that BL-8040, used as a single agent and when combined with pembrolizumab, promoted an increased population of T-cells, activated cytotoxic T-cells, decreased density of MDSCs, resulted in rapid tumor cell apoptosis, and was associated with an improved mean overall survival [125].

Because of the central role that fibroblasts are believed to play in the stromal environment, CAFs are the target of multiple agents. Many such agents specifically target fibroblast activation protein (FAP). For example, sibrotuzumab is a humanized monoclonal anti-FAP antibody, which was evaluated in a single arm study of patients with metastatic colorectal cancer [126]. Although this study demonstrated safety, the efficacy end point was not met, and further studies of this agent have not to our knowledge been performed. Another study of sibrotuzumab published around the same time also demonstrated the safety of this agent [127]. Beyond antibody-based therapeutics, small molecule inhibitors have also been developed to target FAP. Talabostat is one such FAP inhibitor. In a phase II study, talabostat in combination with gemcitabine was found to be safe. However, there was a limited clinical benefit in patients with metastatic PDAC [128]. Furthermore, the use of chimeric antigen receptors (CARs) specific to FAP on T cells has been shown to interrupt the typical desmoplastic function of cancer cells in a mouse model of lung cancer, thereby conferring an anti-tumor effect [129,130]. An ongoing trial is evaluating the safety and efficacy of CAR-T cell therapy specifically targeting FAP (NCT03932565).

Prior studies have also attempted to disrupt the ECM, which contributes to the desmoplastic barrier in the TME of PDAC. A significant component of the ECM is hyaluronan, which is a non- sulfated glycosaminoglycan. In fact, higher levels of tumor hyaluronan have been associated with poorer prognosis among patients with PDAC. Previous studies evaluating the use of hyaluronidase have demonstrated associated improvement in survival in multiple tumor types [131]. A retrospective study associated higher levels of tumor hyaluronan with poorer survival in patients with PDAC [132]. Preclinical studies targeting human recombinant PH20 hyaluronidase (PEGPH20) in a mouse model of PDAC revealed improved vascular permeability and increased drug delivery, which led to an improved effect with standard chemotherapy [133]. Subsequent clinical trials were conducted to evaluate the efficacy of PEGPH20 in combination with standard combined chemotherapy regimens for pancreas cancer. A randomized phase II study of PEGPH20 in combination with gemcitabine and nab-paclitaxel revealed improved progression-free survival without a significant increase in thromboembolic events [134]. However, a randomized phase Ib/II study of PEGPH20 in combination with modified fluorouracil, leucovorin, irinotecan, and oxaliplatin (mFOLFIRINOX) was associated with worse overall survival and increased GI toxicity and thromboembolic events compared to mFOLFIRINOX alone [135]. A follow-up phase III study of PEGPH20 in combination with gemcitabine and nab-paclitaxel revealed lack of improvement of either progression-free or overall survival [136]. Although it is not clear why the addition of PEGPH20 in combination with standard chemotherapy resulted in comparable or worse overall survival, it is possible that increased toxicity leading to dose reduction of chemotherapy may have negatively impacted survival. Furthermore, although the goal of PEGPH20 treatment is to disrupt the ECM in an effort to improve drug delivery, it is possible that ECM disruption may increase the cancer’s ability to metastasize, thereby leading to worse outcomes [137].

FAK inhibition has been shown to modulate the immune milieu and fibrosis within the TME and is accordingly being pursued in clinical studies as a potential therapeutic target along with standard chemotherapy and/or immunotherapy particularly in pancreatic cancer. Increased levels of FAK in the TME have been shown to correlate with poorer prognosis in multiple cancer types [138,139]. The FAK inhibitor defactinib is being tested in multiple tumor types, including in platinum-resistant ovarian cancer in the ROCKIF trial, which is a phase I study evaluating the safety and efficacy of combining defactinib with carboplatin and paclitaxel (NCT03287271). FAK inhibition is also being tested in the phase I setting in non-small cell lung cancer with CT-707, which is a combined FAK/ALK/Pyk2 inhibitor (NCT02695550). Most ongoing clinical trials involving FAK inhibitors in pancreas cancer involve a combined therapeutic approach including immunotherapy. Given the resistant nature of PDAC to systemic therapies and multiple potentially promising treatment strategies, treatment paradigms involving combinations of multiple adjuvant therapies may give the best chance of achieving significant improvement in long term outcomes.

## 7. Combinatorial Systemic Strategies

PDAC remains a challenging disease to treat despite advances in systemic therapy. Currently, the best treatment that results in proven clinical benefit is combination systemic chemotherapy. The phase III MPACT and PRODIGE-4/ACCORD-11 studies unequivocally showed the benefit of combination gemcitabine plus Nab-paclitaxel and FOLFIRINOX vs. single agent gemcitabine, with median survival of 8.5 and 11.1 months, respectively. These chemotherapeutic options represent the frontline agents for metastatic PDAC. Although modern chemotherapy approaches have resulted in improved outcomes, long-term survival remains poor. Combinatorial approaches with chemotherapy, radiation, immunotherapy, and/or targeted therapies may result in improved outcomes for patients with this challenging to treat disease. Given the promise of immunotherapy in improving outcomes in multiple tumor types, many emerging treatment strategies involve immune stimulation as part of the therapeutic approach. The TME generally and the tumor stroma specifically are promising targets for combinatorial therapies in patients with PDAC (Table 2).

Promising preclinical evidence has opened the door for clinical studies investigating FAK inhibitors in combination with chemotherapy and immunotherapy. Preclinical studies have demonstrated that FAK inhibition can potentiate the effect of anti-PD-1 therapy in pancreas cancer [96]. In a phase I study, defactinib, a competitive reversible inhibitor of FAK, was tested along with the PD-1 inhibitor pembrolizumab and gemcitabine in patients with advanced PDAC, and results revealed the combination to be well-tolerated and clinically efficacious due to positive changes in T-cell infiltration (NCT02546531) [140]. A phase II study of defactinib in combination with pembrolizumab is presently being conducted in the setting of high-risk resectable pancreatic cancer (NCT03727880). Furthermore, phase II studies in malignant pleural mesothelioma and ovarian cancer have yielded encouraging results, demonstrating the potential of SMIs in treating aggressive and resilient malignancies such as PDAC. An ongoing clinical trial continues to evaluate the safety and efficacy of defactinib in combination with pembrolizumab in multiple tumor types (NCT02758587).

Multiple preclinical and clinical studies have also demonstrated potential benefit for use of other agents targeting tumor stroma in combination with immunotherapy, such as those that target CXCR2. For example, AZD5069 is a CXCR2 antagonist that blocks neutrophil migration and reduces circulating neutrophil counts. AZD5069 is currently being tested in a phase I/II trial along with gemcitabine/nab-paclitaxel and durvalumab in patients with metastatic PDAC (NCT02583477). Similarly, BMS-813160, which is a CCR2/CCR5 antagonist, is being tested in combination with nivolumab and either irinotecan/5-FU/leucovorin or gemcitabine/nab-paclitaxel in patients with advanced pancreas cancer in an effort toto assess safety and efficacy (NCT03184870).

Another emerging systemic treatment combination involves the use of devimistat, which targets enzymes involved in the mitochondrial tricarboxylic acid (TCA) cycle. These alterations in the TCA induced by devimistat are believed to result in changes in mitochondrial enzyme activity and oxidation/reduction state that lead to apoptosis, necrosis, and autophagy in pancreas cancer cells. A single institution phase I trial of devimistat and FOLFIRINOX demonstrated safety and potential efficacy of this regimen among patients with metastatic pancreatic cancer [141]. A large randomized phase III study investigated the efficacy of using devimistat in addition to FOLFIRINOX in the front line setting for metastatic PDAC patients (NCT03504423). This strategy, the AVENGER 500 study, represented one of the more promising approaches to build upon traditional cytotoxic chemotherapy backbone therapy for PDAC. In Phase I/II trials CPI-613 doubled the historical objective response rate from 31% to 61% with a PFS of 9 months and a median OS of 19 months. This all occurred in the context of little toxicity [142]. Consequently, the FDA granted fast track designation for CPI-613 in November of 2020 [143]. Unfortunately AVENGER 500 did not meet its primary endpoint. CPI-613 in conjunction with FOLFIRINOX showed no significant improvement in OS, with median OS of 11.1 months in treatment arm compared to 11.7 months in FOLFIRINOX only control [144]. These findings should not discourage ongoing or future Phase IIItrials within the same combinatorial strategy.

Combination therapy with radiation and immunotherapy also has a promising role for improving outcomes for patients with PDAC. It has long been recognized that T cells play an important role in the anti-tumor efficacy of radiation therapy as tumor-bearing mice lacking T cells need significantly more RT dose to achieve similar tumor control [145]. Targeting PD-1-PD-L1 in combination with radiation has also revealed significant improvements in tumor response and survival in mouse models of breast cancer [146] and glioma [147]. Multiple ongoing clinical trials are evaluating the role of combination SBRT and immunotherapy with or without chemotherapy for patients with locally advanced PDAC (NCT03245541, NCT04247165, NCT02311361). Furthermore, an ongoing phase III study is evaluating the role of combining algenpantucel-L, an allogeneic pancreatic cancer vaccine, along with adjuvant gemcitabine or 5-FU-based chemoradiation for patients with surgically resected PDAC (NCT01072981).

Additionally, efficacy signals appreciated with PARPi and previous knowledge that DNA damage enhances radiation induced Type I interferon, provided similar rationale for exploring combination with PARPi, RT and immunotherapy. Preclinical studies by Zhang et al., were able to show [148] that in a preclinical model significant tumor growth inhibition with combined treatment with PARPi, olaparib, radiotherapy, and anti-PD-L1 antibody which was significant relative to doublet therapy with olaparib-radiation or anti-PD-L1-radiation, and associated with a 20% complete response rate. This has provided a rationale for ongoing combinatorial clinical trials in this arena (NCT04548752).

Another extremely unique and promising approach in advanced pancreatic cancer is the PANOVA-3 (NCT03377491) study. This clinical trial explores the use of Tumor Treating Fields (TTF) in locally advanced PDAC patients. TTF are electrical currents that alter the spindle apparatus and cytokinesis impacting cell mitosis. In preliminary pre-clinical studies, TTF led to the killing of rapidly dividing cancer cells, including glioblastoma cells and PDAC, where for the former there is already FDA approval [149]. The initial PANOVA-2 trial demonstrated that TTF combined with gemcitabine significantly improved median PFS from 3.7 months among historical controls to 8.3 months and median overall survival (OS) from 6.7 months to 14.9 months [150]. Currently, TTF is being evaluated in combinatorial fashion with gemcitabine and nab-paclitaxel in locally advanced pancreatic cancer.

The most promising strategy for future therapeutic change in the treatment paradigm for pancreatic cancer is the Precision Promise study (NCT04229004). Precision Promise is a multi-center, Phase 2/3 platform trial designed to evaluate multiple regimens in advanced pancreatic cancer. The goal of this novel platform study is to efficiently evaluate multiple experimental therapies compared to standard of care therapy in first and second-line patients. An adaptive design of this study allows for drug approaches that are working to move forward within the context of the study, whereas less efficacious treatment modalities are removed. This is all analyzed in real time across 15 clinical trial sites. Importantly, new arms to this study which can impact cancer cells, modulate the TME and stroma are being added presently. Additionally, this study incorporates translational molecular mining and biomarker exploration to help not only inform therapy testing, but ultimately impact patient outcomes.

## 8. Prospective and Conclusions

Pancreatic adenocarcinoma remains a difficult to treat disease. Traditional forms of therapy including chemotherapy, radiation therapy and surgery though commonly employed in a multimodal fashion still have significant limitations in therapeutic potential for pancreas cancer. As a result, clinical outcomes and survival remain poor. The last two decades have brought about novel and significant advancements in therapeutics across numerous malignancies, chiefly in the domain of immunotherapy, from checkpoint inhibitors to vaccine therapy. Yet the testing of these agents in pancreatic cancer has been met with little clinical success. Promising biologic changes noted on correlative bench science in analyzed tumor specimens treated with novel agents has not consistently translated into clinical success. However, preclinical and clinical work has highlighted that the tumor microenvironment serves as not only a significant hurdle to therapeutic success of novel agents, but also serves as an attractive target for therapy. Immunosuppressive elements, such as myeloid cells, the highly desmoplastic stroma with abundance of cancer-associated fibroblasts or intracellular elements of these cells that promote disease progression, are being consistently evaluated as targets for therapy. There are some important factors to consider in developing novel and hopefully successful therapeutic approaches for pancreatic cancer in the future. First, tumor microenvironment modulation should be considered a critical and adjunctive approach in any treatment paradigm. Secondly, single agent or single therapeutic approaches are unlikely to be met with success. Combinatorial approaches using standard therapies, including surgery or surgical advances for PDAC along with novel small molecular targeting and immunotherapy, are more likely to lead to therapeutic success. Furthermore, combination approaches or agents which simultaneously address the dense stroma and immunomodulation, such as FAK inhibition are even more likely to lead to improved clinical outcomes. Meaningful clinical outcomes may result from combination immunotherapy with stromal targeting agents in pancreas cancer in an effort to overcome immunosuppression in the TME by targeting multiple immunosuppressive pathways simultaneously. Thirdly, sequencing of therapies will also be important to consider in multiagent approaches, as immune and TME priming are important to immunotherapy efficacy in a tumor type considered to be immunologically inactive. Ultimately, novel platform studies, like Precision Promise, which are assessing multiple combinatorial approaches are the most likely to lead to best treatment approach in pancreatic cancer care. Using correlative translational science from these clinical trials looking at novel combinatorial approaches to guide the development of better targeting agents and novel combinations in an iterative fashion will lead to the greatest advances in the treatment of pancreatic ductal adenocarcinoma.

## Figures and Tables

**Table 1:** Summary of Advances in PDAC Treatment Modalities.

Category	Advances
Immunotherapy	Anti-CTLA4 Antibodies
	PD-1/PD-L1 Inhibitors
	CD-40 Agonists
	TME Modulating Vaccines
Targeted Therapy	FAK pathway Inhibition
	CCR2/CCR5 Inhibition
	PARP Inhibition
Stromal Targeting	CXCR4 Inhibition
	FAP Inhibition
	ECM Targeting
Surgical Strategies	IRE
	Appleby
Combinatorial Strategies	Radiation + Immunotherapy +/− Chemotherapy
	Small Molecule Inhibitors + Immunotherapy
	Stromal Targeting + Immunotherapy

**Table 2:** Clinical Trials of Agents Targeting the PDAC Tumor Microenvironment.

Target:	Agents:	Current Clinical Trial:	Notes:
T cell	Nivolumab + nab-paclitaxel +/− gemcitabine	NCT02309177, phase I, (https://clinicaltrials.gov/ct2/show/NCT02309177), accessed on 27 August 2021	Nivolumab is an inhibitor of the immune checkpoint PD-1
	Durvalumab + SBRT	NCT03245541, phase I/II, (https://clinicaltrials.gov/ct2/show/NCT03245541), accessed on 27 August 2021	Durvalumab is an inhibitor of the immune checkpoint PD-1
	Nivolumab + ipilimumab + nab-paclitaxel + gemcitabine + SBRT	NCT04247165, phase I/II, (https://clinicaltrials.gov/ct2/show/NCT04247165), accessed on 27 August 2021	Nivolumab and ipilimumab are inhibitors of the immune checkpoints PD-1 and CTLA4, respectively
	Durvalumab + tremelimumab + nab-paclitaxel + gemcitabine	NCT02658214, phase I, (https://clinicaltrials.gov/ct2/show/NCT02658214), accessed on 27 August 2021	Durvalumab and tremelimumab are inhibitors of the immune checkpoints PD-1 and CTLA4, respectively
	Durvalumab and/or tremelimumab + SBRT	NCT02311361, phase I/II, (https://clinicaltrials.gov/ct2/show/NCT02311361), accessed on 27 August 2021	Durvalumab and tremelimumab are inhibitors of the immune checkpoints PD-1 and CTLA4, respectively
Dendritic cell	CRS-207 + nivolumab + ipilimumab +/− GVAX	NCT03190265, phase II, (https://clinicaltrials.gov/ct2/show/NCT03190265), accessed on 27 August 2021	CRS-207 is a live-attenuated Listeria monocytogenes engineered to express mesothelin, a tumor-associated antigen, and GVAX is a cancer vaccine composed of whole tumor cells genetically modified to secrete the immune stimulatory cytokine, granulocyte-macrophage colony-stimulating factor (GM-CSF)
	Algenpantucel-L	NCT00569387, phase II, (https://clinicaltrials.gov/ct2/show/NCT00569387), accessed on 27 August 2021	Algenpantucel-L is an allogeneic pancreatic cancer vaccine based on the concept of hyperacute rejection and is composed of two human pancreatic ductal adenocarcinoma cell lines (HAPa-1 and HAPa-2)
	Algenpantucel-L	NCT03165188, phase II, (https://clinicaltrials.gov/ct2/show/NCT00569387), accessed on 27 August 2021	
	Adjuvant gemcitabine or 5-F- based chemoradiation +/− Algenpantucel-L	NCT01072981, phase III, (https://clinicaltrials.gov/ct2/show/NCT01072981), accessed on 27 August 2021	
Cancer cell	Olaparib	NCT02184195, phase III, (https://clinicaltrials.gov/ct2/show/NCT02184195), accessed on 27 August 2021	Olaparib is a PARP inhibitor, used as maintenance in this study for patients with known deleterious or suspected deleterious germline BRCA mutation who did not progress on first line platinum-based chemotherapy
	Olaparib +/− pembrolizumab	NCT04548752, phase II, (https://clinicaltrials.gov/ct2/show/NCT04548752), accessed on 27 August 2021	Olaparib is a PARP inhibitor, and pembrolizumab is an inhibitor of the immune checkpoint PD-1
	Niraparib	NCT03601923, phase II, (https://clinicaltrials.gov/ct2/show/NCT03601923), accessed on 27 August 2021	Niraparib is a PARP inhibitor
	Niraparib + ipilimumab or nivolumab	NCT03404960, phase I/II, (https://clinicaltrials.gov/ct2/show/NCT03404960), accessed on 27 August 2021	
	Rucaparib	NCT03140670, phase II, (https://clinicaltrials.gov/ct2/show/NCT03140670), accessed on 27 August 2021	Rucaparib is a PARP inhibitor, used as maintenance in this study for patients with known deleterious or suspected deleterious germline BRCA1/2 or PALB2 mutation who did not progress on first line platinum-based chemotherapy
	Rucaparib	NCT02042378, phase II, (https://clinicaltrials.gov/ct2/show/NCT02042378), accessed on 27 August 2021	
	Pelareorep + pembrolizumab + gemcitabine + irinotecan + leucovorin + 5-FU	NCT02620423, phase I, (https://clinicaltrials.gov/ct2/show/NCT02620423), accessed on 27 August 2021	Pelareorep is an oncolytic reovirus that acts specifically in tumors with an activated Ras pathway
Myeloid	APX005M + nab-paclitaxel + gemcitabine +/− nivolumab	NCT03214250, phase I/II, (https://clinicaltrials.gov/ct2/show/NCT03214250), accessed on 27 August 2021	APX005M is a CD-40 agonist
Cytokine	Plerixafor + cemiplimab	NCT04177810, phase II, (https://clinicaltrials.gov/ct2/show/NCT04177810), accessed on 27 August 2021	Plerixafor is an inhibitor of the alpha chemokine receptor CXCR4, and cemiplimab is an inhibitor of the immune checkpoint PD-1
	BL-8040 + pembrolizumab +/− liposomal irinotecan/5-FU/leucovorin	NCT02826486, phase II, (https://clinicaltrials.gov/ct2/show/NCT02826486), accessed on 27 August 2021	BL-8040 is an inhibitor CXCR4
Stroma	BMS-813160 + nivolumab and/or chemotherapy (irinotecan/5-FU/leucovorin or gemcitabine/nab-paclitaxel)	NCT03184870, phase I/II, (https://clinicaltrials.gov/ct2/show/NCT03184870), accessed on 27 August 2021	BMS-813160 is a CCR2/CCR5 antagonist
	Durvalumab + AZD5069 or gemcitabine/nab-paclitaxel	NCT02583477, phase I/II, (https://clinicaltrials.gov/ct2/show/NCT02583477), accessed on 27 August 2021	AZD5069 is a CXCR2 antagonist that blocks neutrophil migration and reduces circulating neutrophil counts
	CCX872-B + oxaliplatin/ irinotecan/5-FU/leucovorin	NCT02345408, phase I, (https://clinicaltrials.gov/ct2/show/NCT02345408), accessed on 27 August 2021	CCX872-B is a CXCR2 antagonist
	PF-04136309 + gemcitabine/nab-paclitaxel	NCT02732938, phase I/II, (https://clinicaltrials.gov/ct2/show/NCT02732938), accessed on 27 August 2021	PF-04136309 is a CXCR2 antagonist
	CAR-T cells targeting Nectin4/FAP	NCT03932565, phase I, (https://clinicaltrials.gov/ct2/show/NCT03932565), accessed on 27 August 2021	CAR-T cell therapy specifically targeting fibroblast activation protein (FAP)
	Defactinib +/− pembrolizumab	NCT03727880, phase I/II, (https://clinicaltrials.gov/ct2/show/NCT03727880), accessed on 27 August 2021	Defactinib is a focal adhesion kinase (FAK) inhibitor
	Defactinib + pembrolizumab + gemcitabine	NCT02546531, phase I/II, (https://clinicaltrials.gov/ct2/show/NCT02546531), accessed on 27 August 2021	
	Defactinib + pembrolizumab	NCT02758587, phase I, (https://www.clinicaltrials.gov/ct2/show/NCT02758587), accessed on 27 August 2021	
Other	Various agents (Multiple Regimes)	NCT04229004, phase II/III, (https://clinicaltrials.gov/ct2/show/NCT04229004), accessed on 27 August 2021	Percision Promise
